# Supplementary material for: mTOR-Dependent Autophagy Regulates Slit Diaphragm Density in Podocyte-like Drosophila Nephrocytes
Source: Cells. 2022 Jul 2;11(13):2103. doi: 10.3390/cells11132103 (PMC9265458; doi:10.3390/cells11132103)
Supplement: Supplementary file 1 [file cells-11-02103-s001.zip › cells-1774655-supplementary.pdf]

Article

mTOR-Dependent Autophagy Regulates Slit Diaphragm Density in Podocyte-like *Drosophila* Nephrocytes

Dominik Spitz [1,†], Maria Comas [1,†,*], Lea Gerstner [1], Séverine Kayser [1], Martin Helmstädter [1], Gerd Walz [1,2] and Tobias Hermle [1,*]

1 Renal Division, Department of Medicine, Faculty of Medicine and Medical Center; University of Freiburg, 79106 Freiburg, Germany; dominik.spitz@uniklinik-freiburg.de (D.S.); lea.gerstner@uniklinik-freiburg.de (L.G.); severine.kayser@uniklinik-freiburg.de (S.K.); martin.helmstaedter@uniklinik-freiburg.de (M.H.); gerd.walz@uniklinik-freiburg.de (G.W.)

2 CIBSS – Centre for Integrative Biological Signalling Studies; 79106 Freiburg; Germany

* Correspondence: tobias.hermle@uniklinik-freiburg.de (T.H.); maria.comas.soberats@uniklinik-freiburg.de (M.C.); Tel.: +49-761-270-33630 (T.H.); +49-0761-270-63046 (M.C.)

† These authors contributed equally to this work.

Supplementary Materials

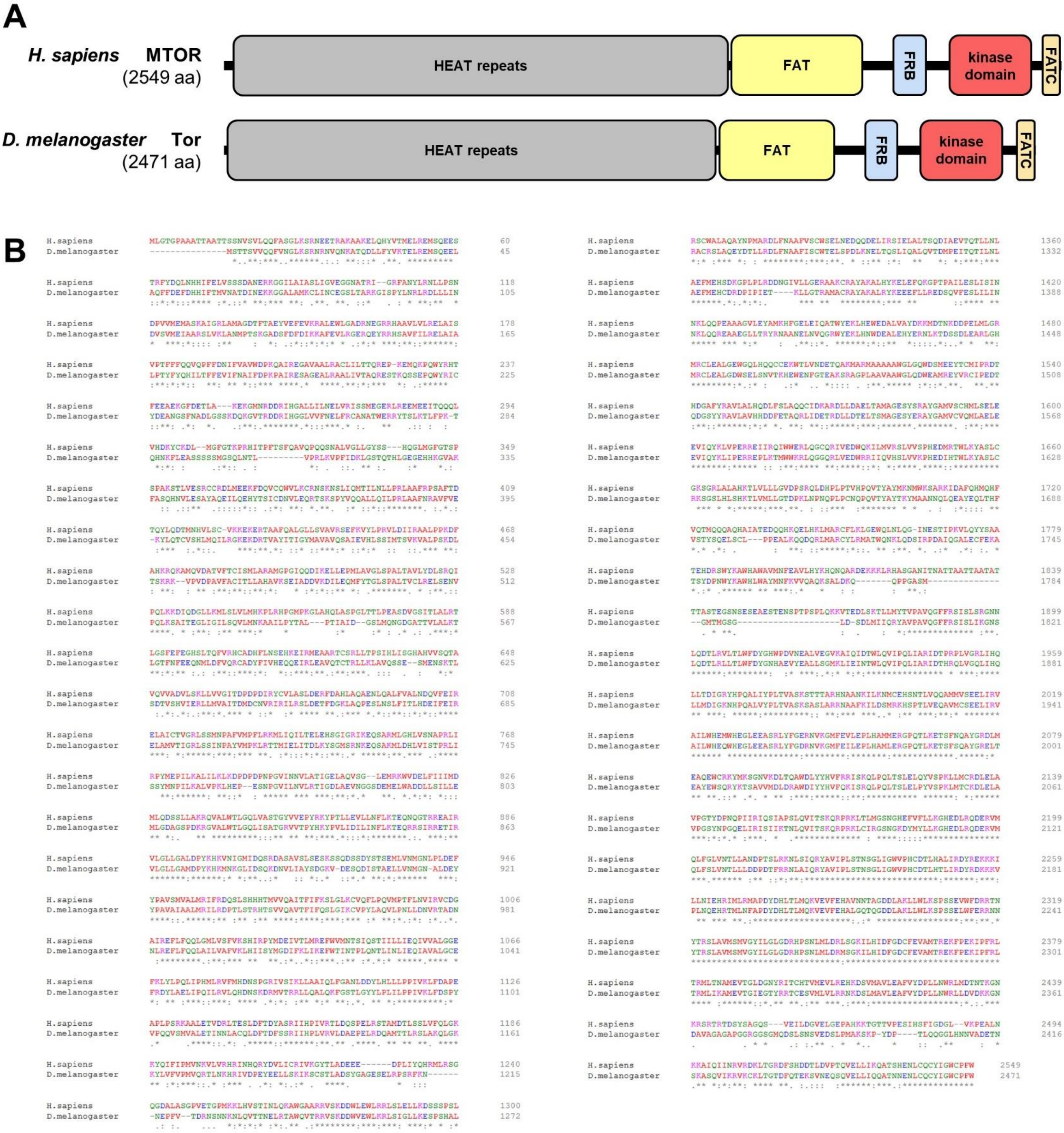

Supplementary Figure S1. The Tor protein is evolutionary conserved from *Homo sapiens* to *Drosophila*. (**A**) Schematic represents the human and *Drosophila* Tor proteins and their functional domains. (**B**) Alignment of the amino acid sequences of human MTOR and *Drosophila* Tor proteins using Clustal Omega (ebi.ac.uk/Tools/msa/clustalo/).

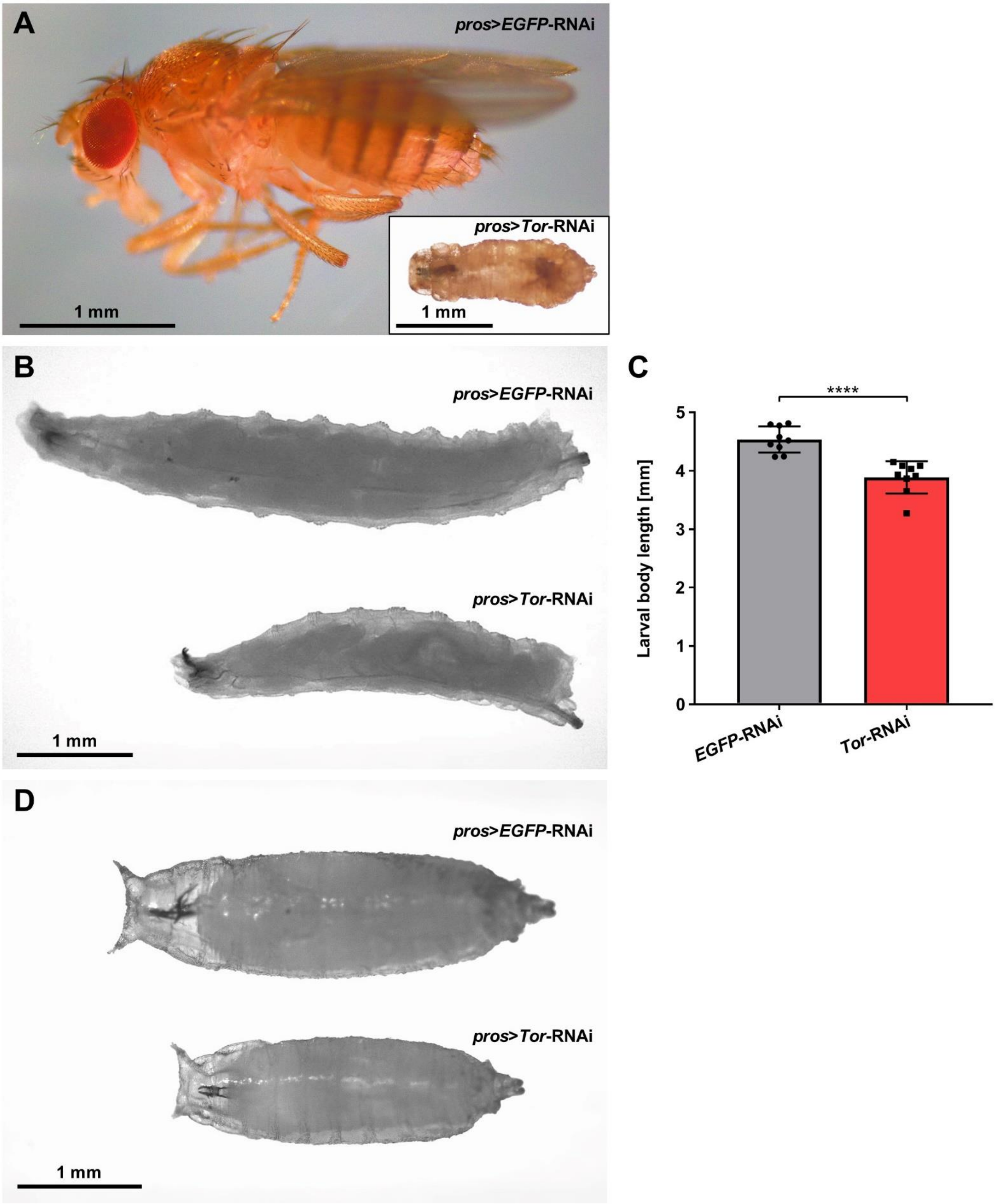

Supplementary Figure S2. Tor inhibition results in decreased larval body length and pupal lethality. (**A**) Flies expressing the control RNAi develop normally (representative female animal is shown). In contrast, animals expressing *Tor*-RNAi may reach puparium, but die during this stage and do not eclose (see inset). (**B**) Late (wandering) third instar *Drosophila* larvae are shown. The body length of larvae expressing *Tor*-RNAi (bottom) under control of *pros-GAL4* is shorter compared to larvae expressing a control RNAi (top). (**C**) Quantification of larval body length in conditions analogous to (B). Data shows mean ± standard deviation, n=9 animals per genotype. Statistical difference was assessed by unpaired t test, $p<0.0001$ for *Tor*-RNAi. (**D**) *Drosophila* pupae are shown. Length of pupal case for animals expressing *Tor*-RNAi (bottom) is shorter compared to animals expressing a control RNAi (top).

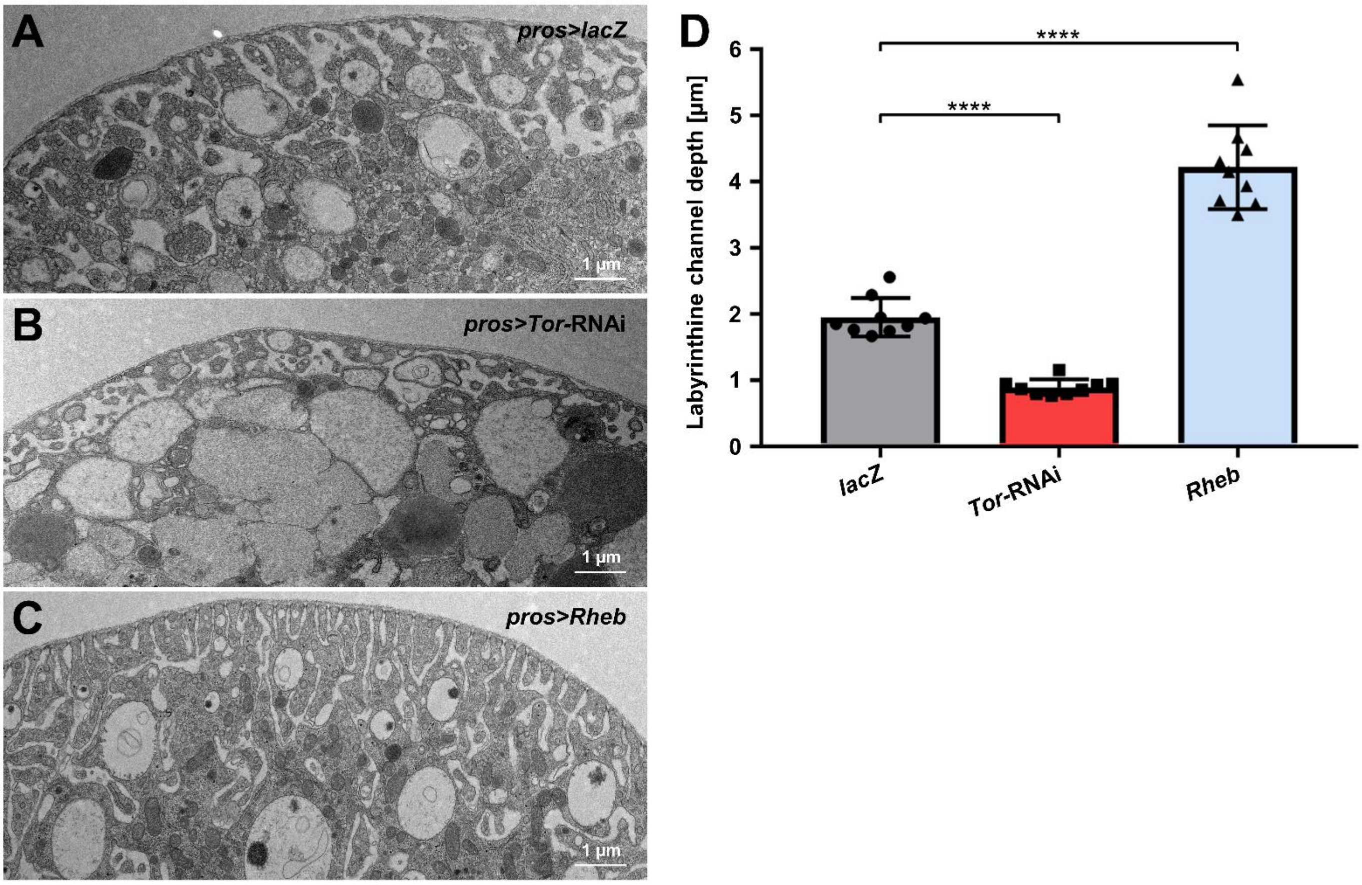

Supplementary Figure S3. Nephrocyte labyrinthine channel depth is positively controlled by mTOR signaling. Transmission electron microscopy (TEM) images of cells expressing *lacZ* (**A**), *Tor*-RNAi (**B**) or overexpressing *Rheb* (**C**). Depth of labyrinthine channels is reduced upon mTOR inhibition. Conversely, channels are deeper when mTOR signaling is induced. (**D**) Quantification of labyrinthine channel depth in (A-C) based on nine representative measurements. Data shows mean ± standard deviation, n=1 animal per genotype with 1 cell each animal. Statistical differences were assessed by one-way ANOVA with post-hoc analysis, $p<0.0001$ for *Tor*-RNAi, $p<0.0001$ for *Rheb*.

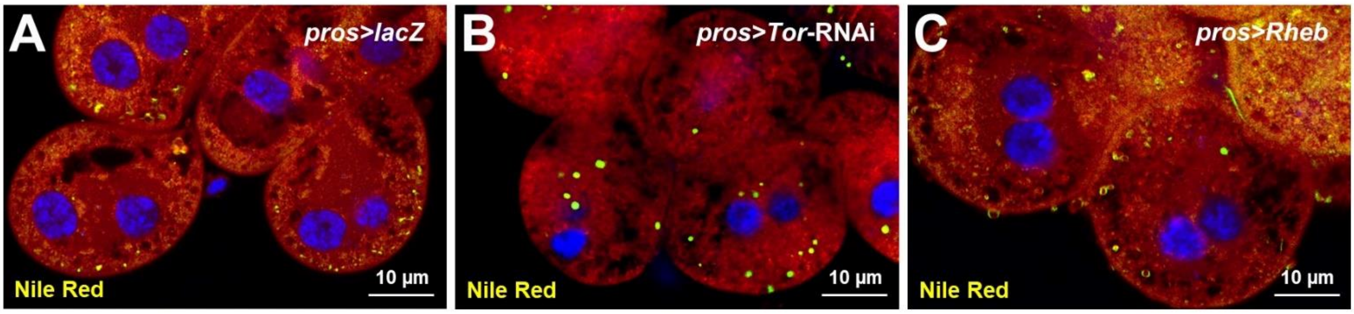

Supplementary Figure S4. Nile Red staining of nephrocytes upon mTOR manipulation. Confocal images of garland cell nephrocytes are shown after Nile Red exposure. This lipophilic dye emits red fluorescence upon binding polar lipids and green/yellow for neutral lipids. The abundance of Nile Red-positive vesicles mildly expands upon mTOR inhibition (**B**), but vesicles remain sparse compared to control (**A**) and gain of mTOR signaling (**C**).

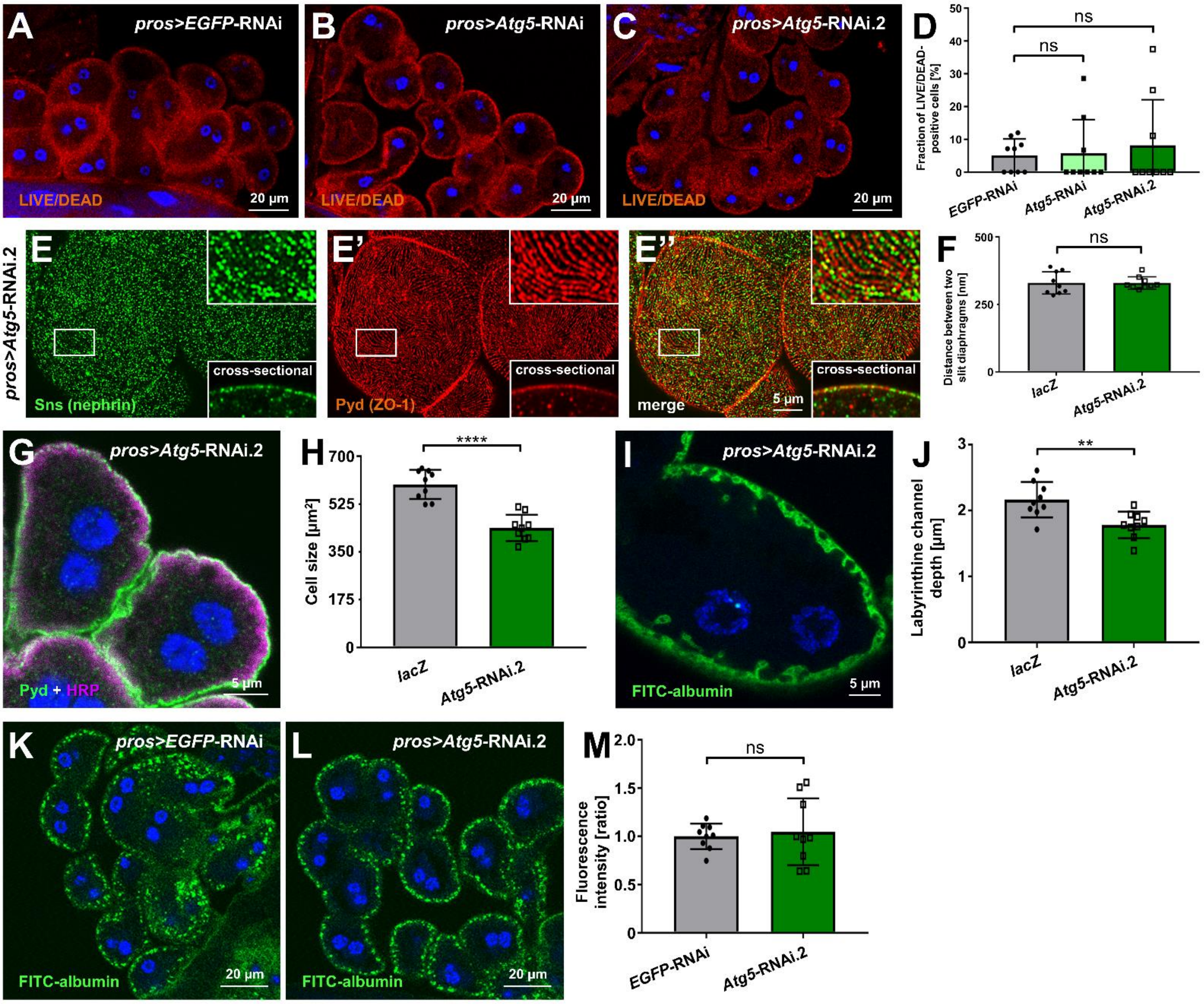

Supplementary Figure S5. A second *Atg5*-RNAi confirms the effects of autophagy in *Drosophila* nephrocytes. (**A-C**) The LIVE/DEAD fixable dye is excluded from viable cells, but in dead cells with an impaired membrane barrier, the dye accumulates. This results in a brighter fluorescence in particular within the nucleus. Numbers of LIVE/DEAD positive cells do not significantly differ among cells expressing *Atg5*-RNAi (**B**) or *Atg5*-RNAi.2 (**C**) compared to cells expressing a control RNAi (**A**). (**D**) Quantification of the fraction of LIVE/DEAD-positive cells in conditions analogous to (A-C). Data shows mean ± standard deviation, n=9 animals per genotype with 14-19 cells on average for each animal. Statistical differences were assessed by one-way ANOVA with post-hoc analysis, $p>0.05$ for *Atg5*-RNAi, $p>0.05$ for *Atg5*-RNAi.2. (**E**) Nephrocytes were co-stained for Sns (nephrin) and Pyd (ZO-1). Magnified regions of the tangential section are shown in the upper insets, surface details from cross sections in the lower insets. Cells expressing *Atg5*-RNAi.2 under control of *pros-GAL4* display the regular slit diaphragm fingerprint pattern of control cells expressing *lacZ* (Figure 1C-C''). (**F**) Quantification of the distance between two slit diaphragms is shown analogous to conditions in (E-E'' and Figure 1C-C''). Distances were measured along a linear path representing the widest diameter of individual cells. Data shows mean ± standard deviation, n=9 animals per genotype with 3 cells each animal. Statistical difference was assessed by unpaired t test, $p>0.05$ for *Atg5*-RNAi.2. (**G**) Nephrocytes were stained for Pyd to assess cell size. Co-staining with HRP confirms correct localization of Pyd at the cell membrane. Cell size is smaller in cells expressing *Atg5*-RNAi.2 compared to *lacZ* expressing control cells (Figure 2E). (**H**) Quantification of cell size by measurement of the area outlined by Pyd staining in the cross-sectional plane in conditions analogous to (G and Figure 2E). Data shows mean ± standard deviation, n=9 animals per genotype with 3 cells each animal. Statistical difference was assessed by unpaired t test, $p<0.0001$ for *Atg5*-RNAi.2. (**I**) Nephrocyte labyrinthine channels are visualized by passive FITC-albumin tracer diffusion into the channels after brief fixation. Channels are shortened in cells expressing *Atg5*-RNAi.2 compared to *lacZ* expressing control cells (Figure 3E). (**J**) Quantification of labyrinthine channel depth analogous to conditions in (I and Figure 3E) based on three representative measurements in sections of the cell surface that are not in immediate proximity to a neighboring cell. Data shows mean ± standard deviation, n=9 animals per genotype with 3 cells each animal. Statistical difference was assessed by unpaired t test, $p<0.01$ for *Atg5*-RNAi.2. (**K-L**) Nephrocyte

function is visualized by the FITC-albumin endocytosis assay. The expression of *Atg5*-RNAi.2 (**L**) does not alter tracer uptake compared to control cells (**K**). (**M**) Quantification of FITC-albumin-derived fluorescence intensity is normalized to the average of an *EGFP*-RNAi expressing control experiment performed in parallel and shown for the indicated genotypes (K-L). Data shows mean ± standard deviation as average of the three brightest representative cells from one animal, n=9 animals per genotype. Statistical difference was assessed by unpaired t test, $p>0.05$ for *Atg5*-RNAi.2.
